# Supplementary material for: Chiral inversion induced by aromatic interactions in short peptide assembly
Source: Nat Commun. 2024 Jul 23;15:6186. doi: 10.1038/s41467-024-50448-0 (PMC11266598; doi:10.1038/s41467-024-50448-0)
Supplement: Supplementary file 1 — Supplementary Information [file 41467_2024_50448_MOESM1_ESM.pdf]

Supplementary Information

Chiral inversion induced by aromatic interactions in short peptide assembly

Kai Qi,[1] Hao Qi,[1] Muhan, Wang,[2] Xiaoyue Ma,[1] Yan Wang,[1] Qiang Yao,[1] Wenliang Liu,[1] Yurong Zhao,[1] Jiqian Wang,[1] Yuefei Wang,[3,*] Wei Qi,[3] Jun Zhang,[4,*] Jian R. Lu,[5,*] Hai Xu[1,*]

[1] State Key Laboratory of Heavy Oil Processing and Department of Biological and Energy Chemical Engineering, China University of Petroleum (East China), 66 Changjiang West Road, Qingdao 266580, China.

[2] Department of Civil Engineering, Qingdao University of Technology, Qingdao 266033, China.

[3] State Key Laboratory of Chemical Engineering, School of Chemical Engineering and Technology, Tianjin University, Tianjin 300072, China.

[4] School of Material Science and Engineering, China University of Petroleum (East China), Qingdao 266580, China.

[5] Biological Physics Group, Department of Physics and Astronomy, The University of Manchester, Manchester M13 9PL, United Kingdom.

CORRESPONDING AUTHOR E-mails: wangyuefei@tju.edu.cn

zhangjunupc@upc.edu.cn

j.lu@manchester.ac.uk

xuh@upc.edu.cn

1. Supplementary method 1

The core-valence bifurcation (CVB) index is a method to examine the strength of hydrogen bonds, based on the topological analysis of the electron localization function (ELF)[1]. The lower the CVB index is, the stronger the H-bond will be. Normal hydrogen bonds can be written as D-H···A, where D is the hydrogen bond donor atom and A the hydrogen bond acceptor atom. The CVB index is defined as:

$$\mathrm{CVB_{index}} = \mathrm{ELF(C\text{-}V)} - \mathrm{ELF(DH\text{-}A)} \tag{1}$$

where ELF(C-V) denotes the bifurcation point value between the core basin and the valence basin, and ELF(DH-A) represents the bifurcation point value between V(D,H) and V(A), that is, the ELF value of the type (3,-1) ELF critical point between H and A atoms.

2. Supplementary method 2

MD simulations of single molecules were performed to generate an array of molecular conformations for each peptide, as described previously[2]. For the initial configuration, there was a single peptide molecule and 8400 H_2O molecules in a water-filled box with a dimension of 64 Å × 64 Å × 64 Å. MD simulations were performed by using the large-scale atomic/molecular massively parallel simulator (LAMMPS) software[3]. Inter- and intra-atomic interactions were described by the all-atom (AA) force field of the optimized potentials for liquid simulations (OPLS-AA) force field, which were composed of pairwise and bonding interactions[4,5]. The SPC/E model was used to describe water molecules[6]. The OPLS force field is expressed as:

$$E_{\text{total}} = E_{\text{bonds}} + E_{\text{angles}} + E_{\text{dihedrals}} + E_{\text{nonbonded}} \quad (2)$$

$$E_{\text{bonds}} = \sum_{\text{bonds}} K_r (r - r_0)^2 \quad (3)$$

$$E_{\text{angles}} = \sum_{\text{angles}} K_\theta (\theta - \theta_0)^2 \quad (4)$$

$$E_{\text{dihedrals}} = \sum_{\text{dihedrals}} \left(\frac{V_1}{2}[1 + \cos(\varphi - \varphi_1)] + \frac{V_2}{2}[1 - \cos(2\varphi - \varphi_2)] + \frac{V_3}{2}[1 + \cos(3\varphi - \varphi_3)] + \frac{V_4}{2}[1 - \cos(4\varphi - \varphi_4)] \right) \quad (5)$$

$$E_{\text{nonbonded}} = \sum_{i>j} \left[\varepsilon_{ij} \left(\frac{\sigma^{12}}{r_{ij}^{12}} - \frac{\sigma^6}{r_{ij}^6} \right) + \frac{q_i q_j e^2}{4\pi\varepsilon_0 r_{ij}} \right] \quad (6)$$

where E_{total} is the total energy in the system which is equal to the energy of bond stretching (E_{bonds}) plus angles shake (E_{angles}) plus dihedrals shake ($E_{\text{dihedrals}}$) plus pairwise ($E_{\text{nonbonded}}$).

The whole MD simulation process comprised two steps. First, a 1 ns NPT ensemble was performed at 298 K and 0.1 MPa to obtain a reliable system density. Then, 2 ns simulations in NVT ensemble were performed for sampling various peptide conformations. The temperature and the pressure were controlled by the Nosé thermostat and barostat, respectively[7,8]. The long-range electrostatic interactions were treated using the PPPM summation[9]. The time step was set as 1 fs. Full trajectories were saved and the frames were output every 1 ps for sampling the results. All the snapshots were displayed using the VMD software[10].

3. Supplementary figures

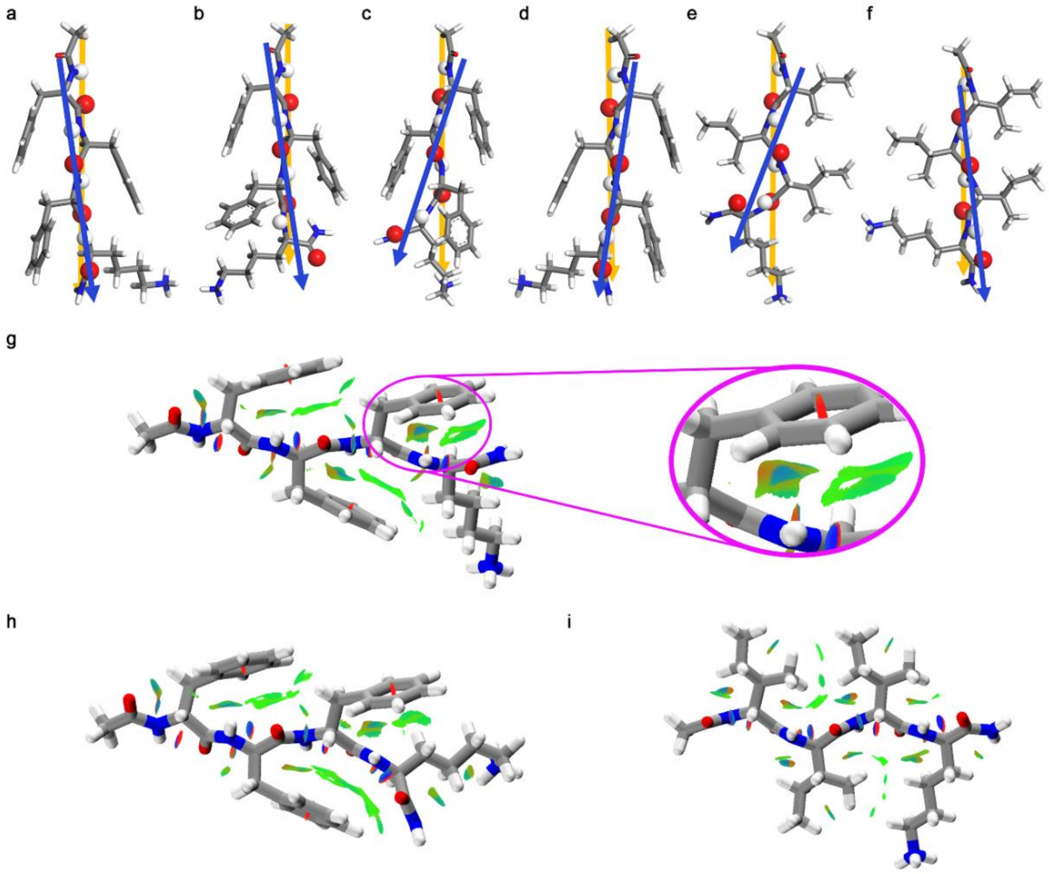

Supplementary Fig. 1 | Single strand conformations, handedness, and intrastrand interactions. a-f Twisting directions of single β-strands for positively charged (**a**) $^{L}F_3{}^{L}K$, (**b**) $^{L}F_3{}^{D}K$, (**c**) $^{D}F_3{}^{L}K$, (**d**) $^{D}F_3{}^{D}K$, (**e**) $^{D}I_3{}^{L}K$, and (**f**) $^{D}I_3{}^{D}K$. **g-i** RDG isosurfaces of single charged $^{D}F_3{}^{D}K$, $^{D}F_3{}^{L}K$, and $^{D}I_3{}^{D}K$ β-strands, respectively. The Lys side chains in these strands were assumed to be 100% protonated and thus carried a positive charge. Atoms coloring scheme is: red, oxygen; blue, nitrogen; white, hydrogen, and gray, carbon. The two arrowed lines (from N- to C-terminus: blue and yellow) connecting potential H-bonding atoms on each side can define the twisting directions of the single strands. The blue, green, and red areas in the RDG isosurfaces represent attractive H-bonding, van der Waals interactions, and steric repulsion, respectively.

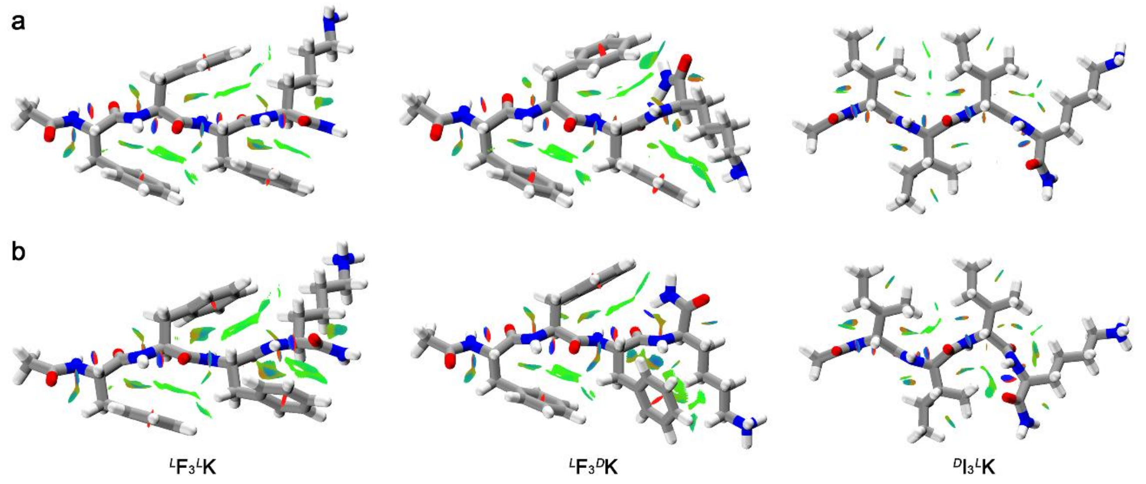

Supplementary Fig. 2 | RDG isosurfaces for single $^{L}F_3{}^{L}K$, $^{L}F_3{}^{D}K$, $^{D}I_3{}^{L}K$ strands. a neutral and **b** positively charged states, respectively. Atoms coloring scheme is: red, oxygen; blue, nitrogen; white, hydrogen, and gray, carbon. The blue, green, and red areas within the RDG isosurfaces represent attractive H-bonding, van der Waals interactions, and steric repulsion, respectively.

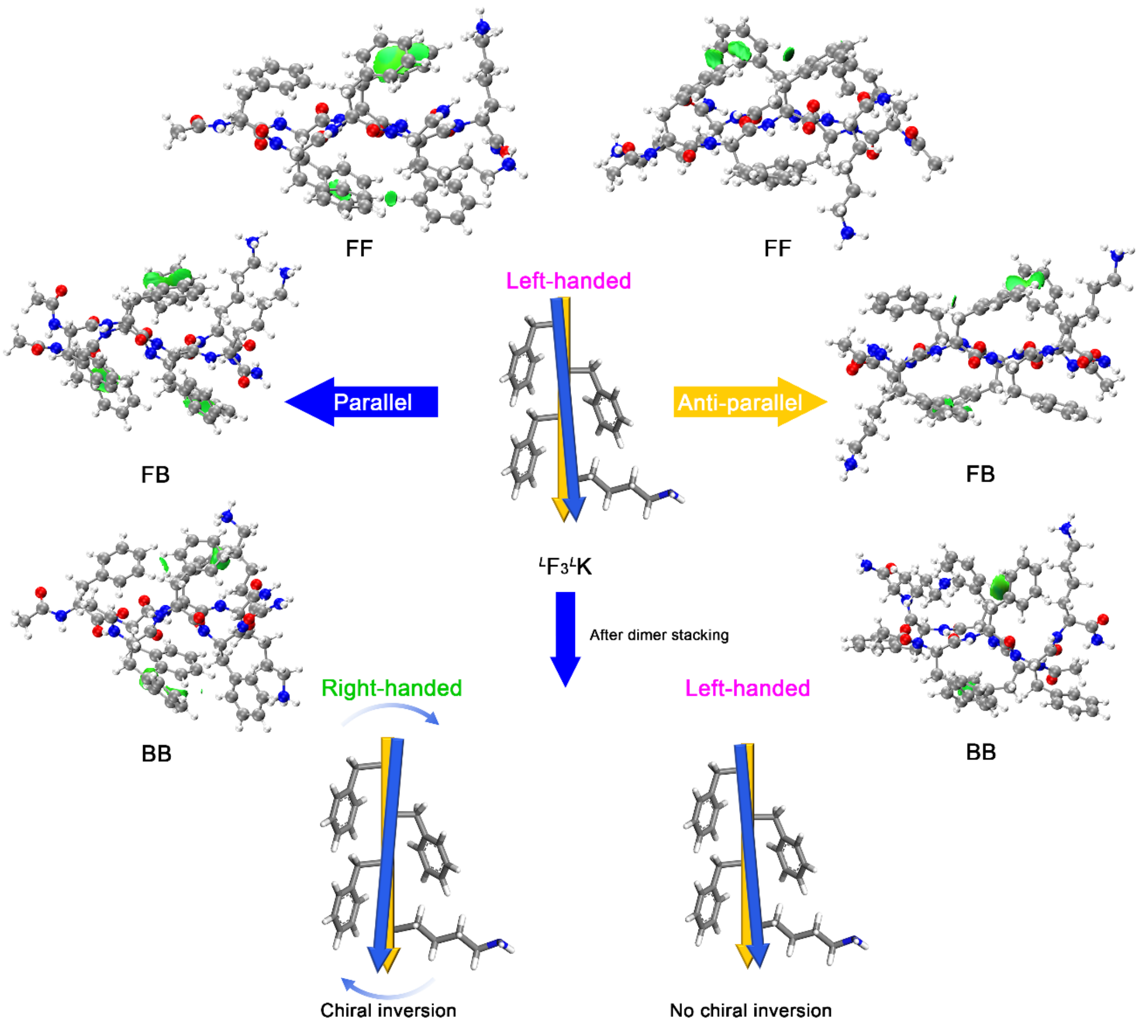

Supplementary Fig. 3 | Dimer conformations, π-π stacking interactions between β-strands, and chiral inversion. IGMH analysis of π-π stacking interactions between β-strands within parallel and anti-parallel dimers for positively charged $^{L}F_3{}^{L}K$. These dimers were obtained after QC structural optimization, based on the single strand conformations described in Supplementary Fig. 1. Compared to the anti-parallel dimers, more extensive and stronger π-π stacking interactions were revealed within the parallel ones, as denoted by green patches in the IGMH isosurfaces. The chiral inversion of single strands only occurred within the parallel dimers. Atoms coloring scheme is: red, oxygen; blue, nitrogen; white, hydrogen, and gray, carbon.

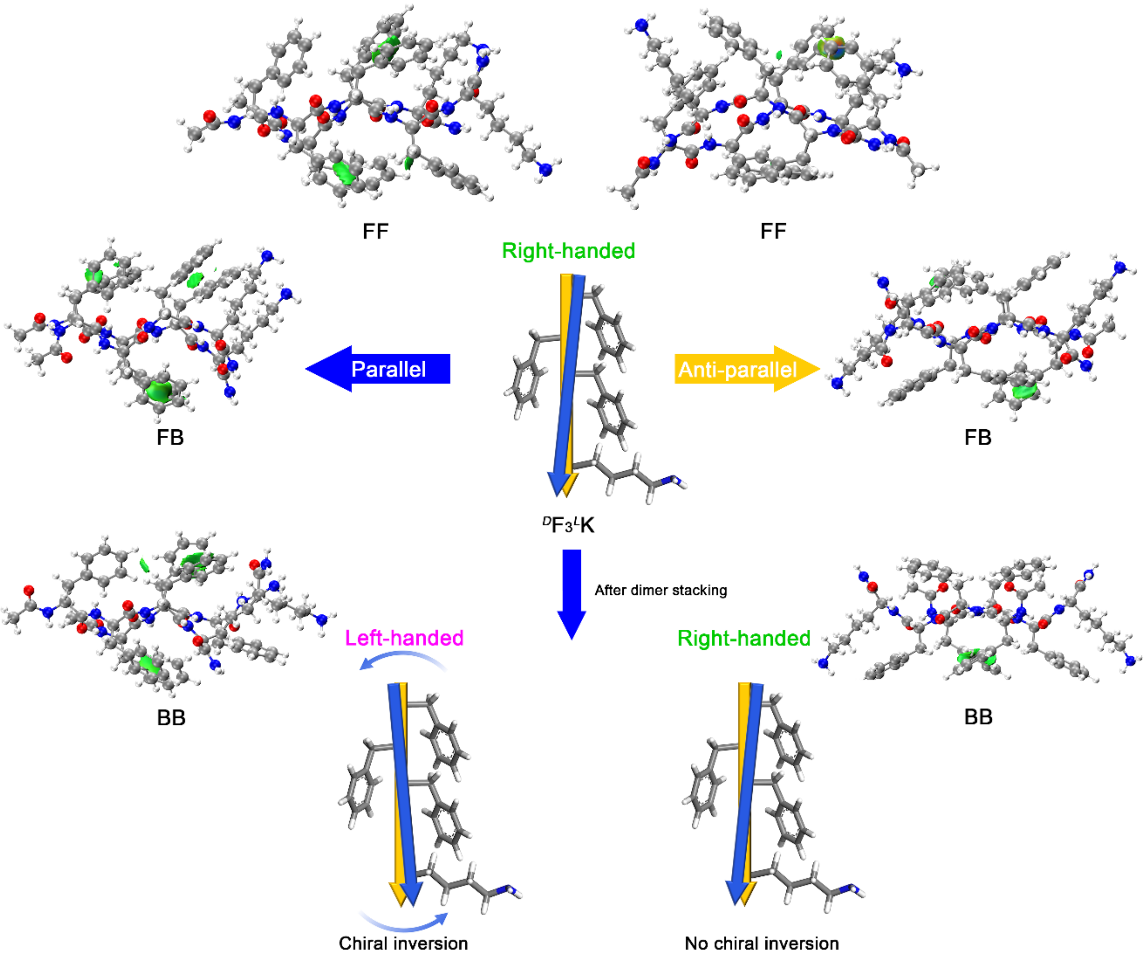

Supplementary Fig. 4 | Dimer conformations, π-π stacking interactions between β-strands, and chiral inversion. IGMH analysis of π-π stacking interactions between β-strands within parallel and anti-parallel $^{D}F_3{}^{L}K$ dimers. These dimers were obtained after QC structural optimization, based on the single strand conformations described in Fig. 1 of the main text. Compared to the anti-parallel dimers, more extensive and stronger π-π stacking interactions were revealed within the parallel ones, as denoted by green patches in the IGMH isosurfaces. Importantly, chiral inversion of single strands only happened within the parallel dimers, irrespective of the contact modes between strands. Atoms coloring scheme is: red, oxygen; blue, nitrogen; white, hydrogen, and gray, carbon.

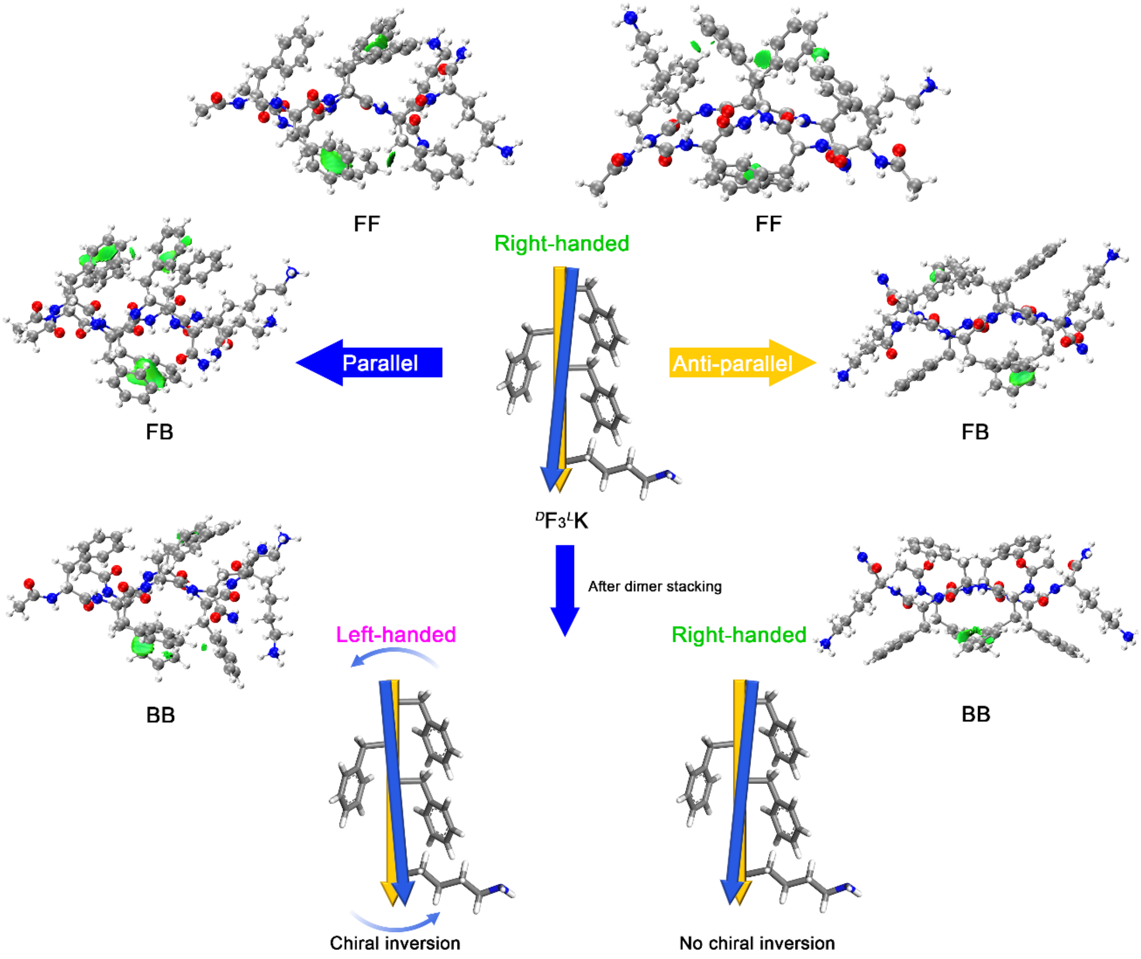

Supplementary Fig. 5 | Dimer conformations, π-π stacking interactions between β-strands, and chiral inversion. IGMH analysis of π-π stacking interactions between β-strands within parallel and anti-parallel dimers for positively charged $^{D}F_3{}^{L}K$. These dimers were obtained after QC structural optimization, based on the single strand conformations described in Supplementary Fig. 1. Compared to the anti-parallel dimers, more extensive and stronger π-π stacking interactions were revealed within the parallel ones, as denoted by green patches in the IGMH isosurfaces. Importantly, chiral inversion of single strands only happened within the parallel dimers, irrespective of the contact modes between strands. Atoms coloring scheme is: red, oxygen; blue, nitrogen; white, hydrogen, and gray, carbon.

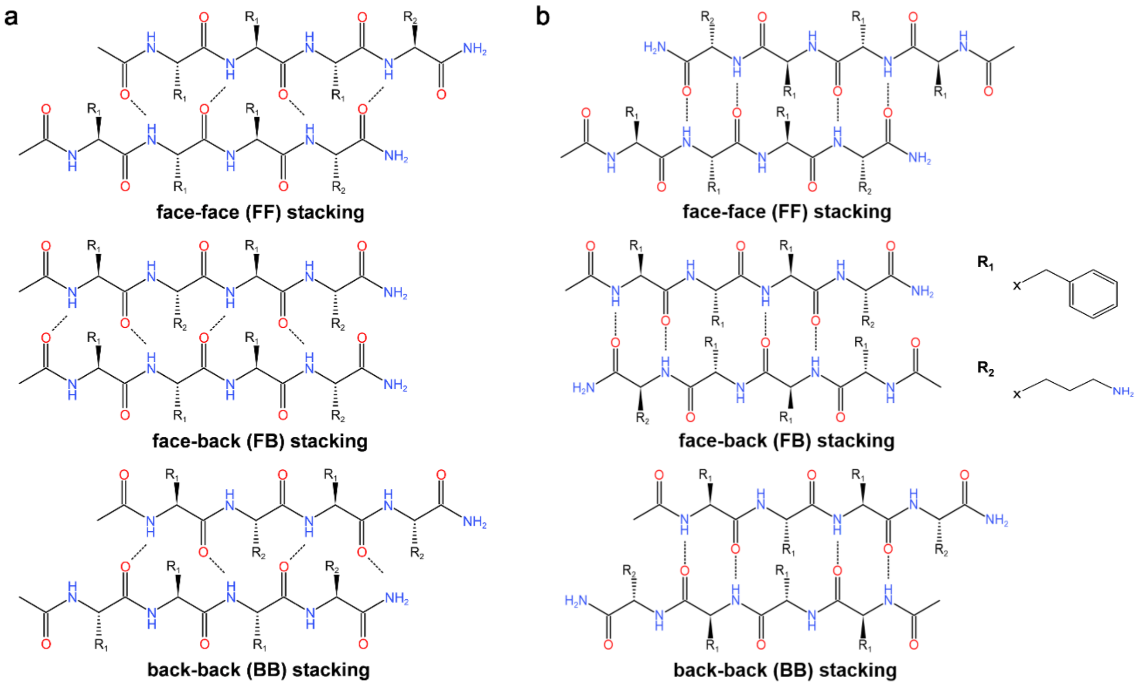

Supplementary Fig. 6 | Strand-strand contacting modes. a parallel and **b** anti-parallel dimers.

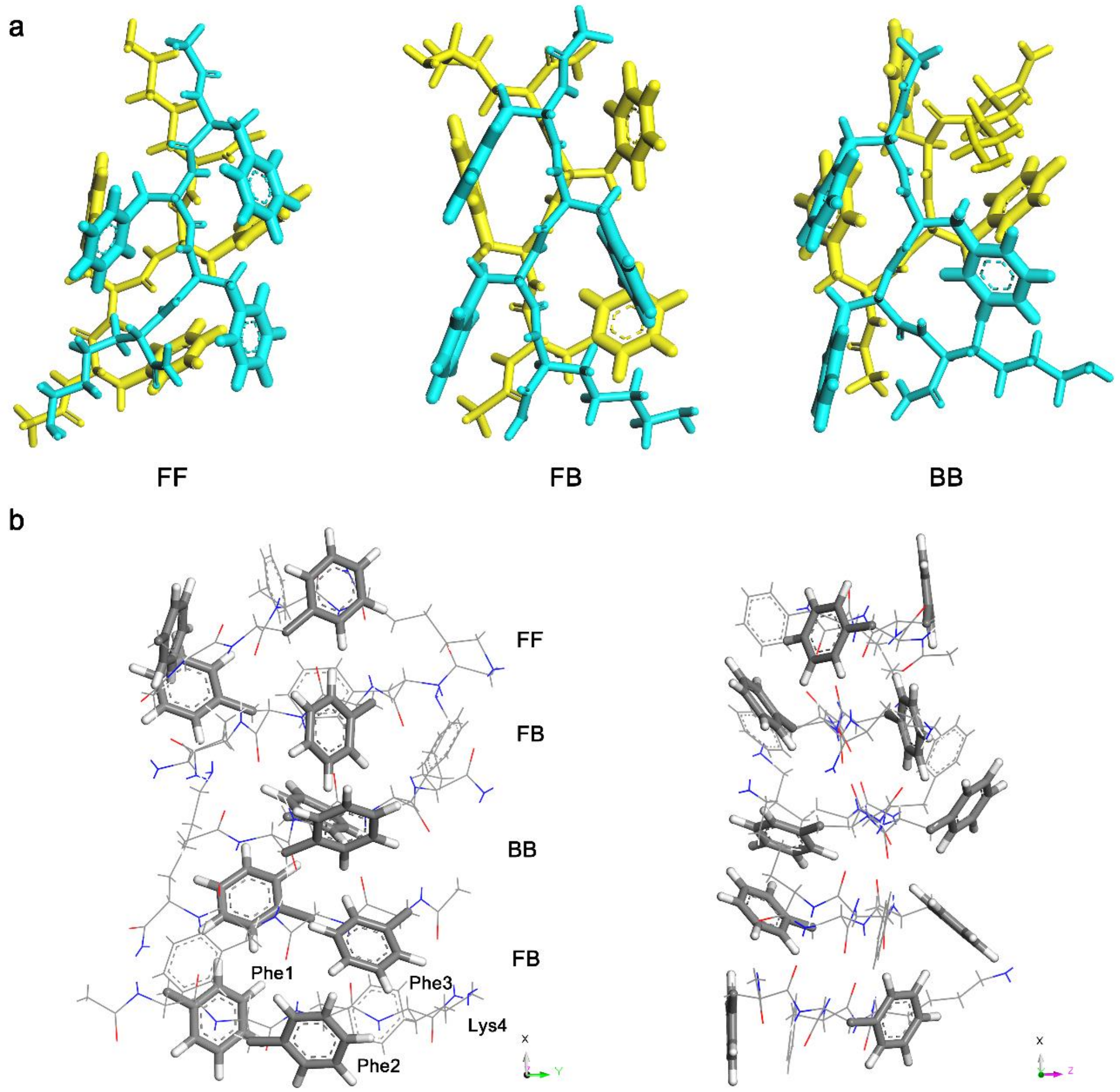

Supplementary Fig. 7 | Anti-parallel $^{L}F_3{}^{L}K$ dimers and pentamer. a Anti-parallel $^{L}F_3{}^{L}K$ dimers with FF, FB, and BB contacting modes. Yellow and blue colours are used to distinguish the two molecules. **b** Anti-parallel $^{L}F_3{}^{L}K$ pentamer containing one FF, one BB, and two FB modes. Atoms coloring scheme is: red, oxygen; blue, nitrogen; white, hydrogen, and gray, carbon.

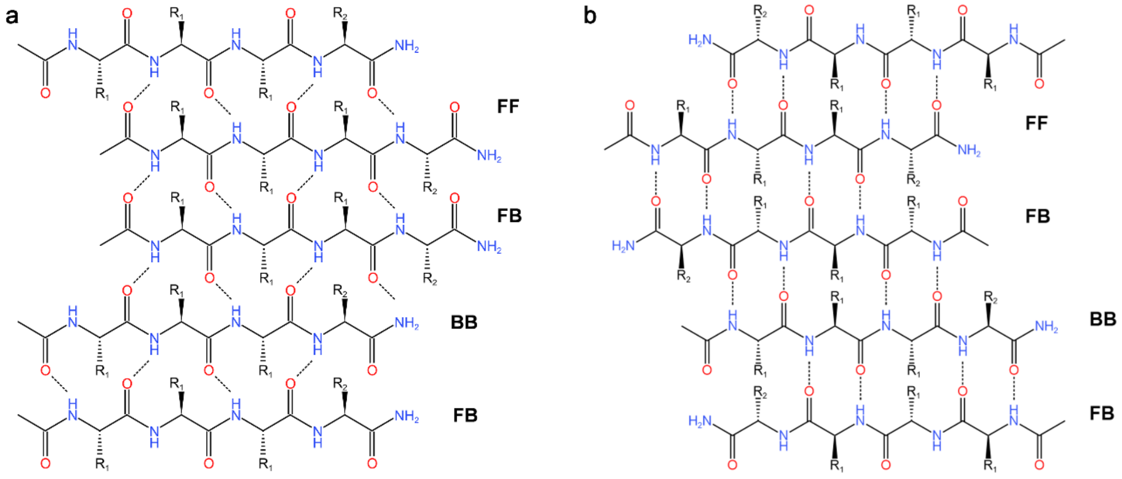

Supplementary Fig. 8 | Contacting modes within $^{L}F_3{}^{L}K$ β-sheet pentamers. a Parallel and **b** anti-parallel arrangements.

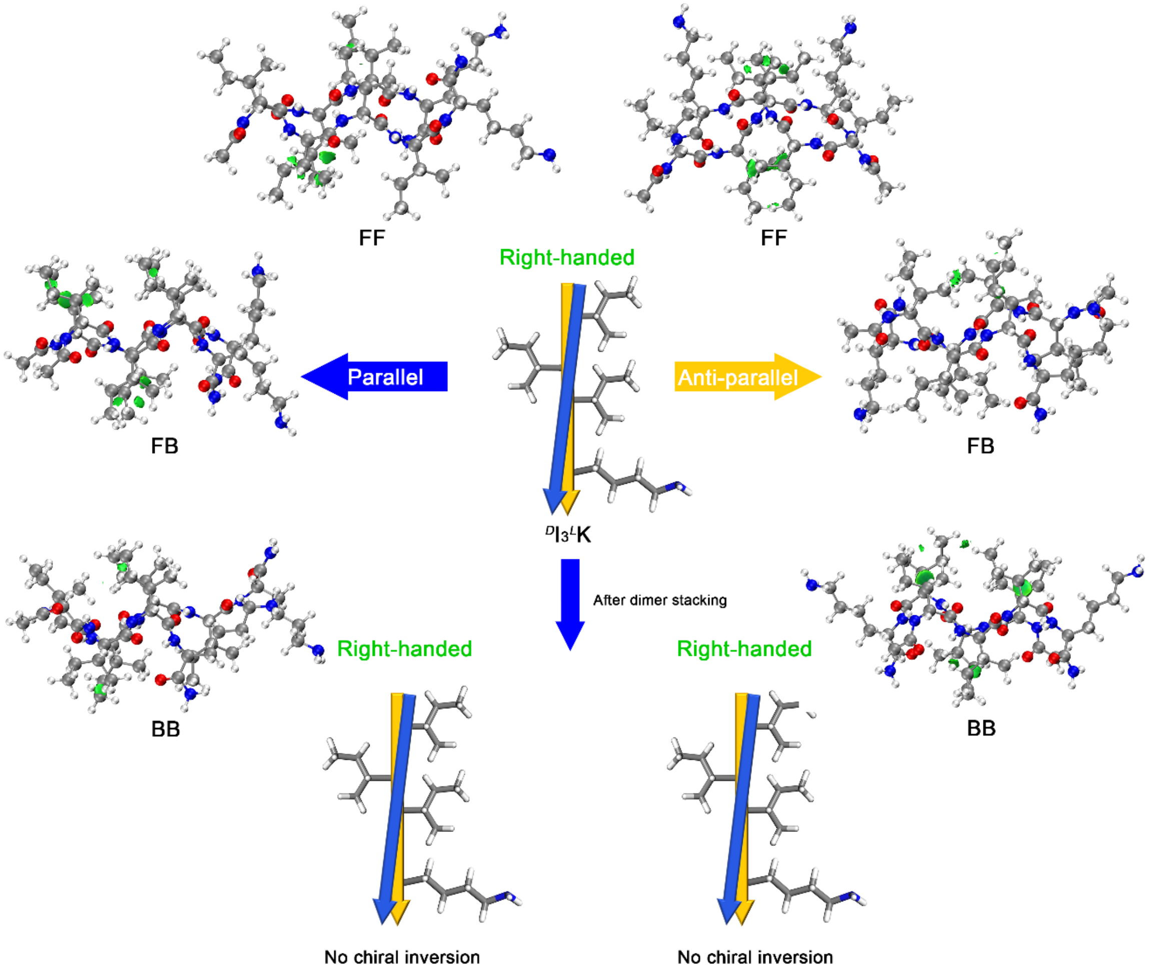

Supplementary Fig. 9 | $^{D}I_3{}^{L}K$ dimer conformations, side chain-side chain interactions between β-strands. IGMH analysis of side chain-side chain interactions between β-strands within parallel and anti-parallel $^{D}I_3{}^{L}K$ dimers. These dimers were obtained after QC structural optimization, based on the single strand conformations described in Fig. 1 of the main text. Atoms coloring scheme is: red, oxygen; blue, nitrogen; white, hydrogen, and gray, carbon. The green regions in the IGMH isosurfaces denote weak van der Waals interactions.

4. Supplementary table

Supplementary Table 1. Binding energies of the dimers of neutral and positively charged $^{L}F_3{}^{L}K$, $^{L}F_3{}^{D}K$, $^{D}F_3{}^{L}K$, $^{D}F_3{}^{D}K$, $^{D}I_3{}^{L}K$ and $^{D}I_3{}^{D}K$ with different arrangements and contacting modes. The values for the charged dimers are shown in parentheses.

Dimer		FF (kcal/mole)	FB (kcal/mole)	BB (kcal/mole)
$^{L}F_3{}^{L}K$	Parallel	-67.74 (-44.03)	-64.56 (-42.10)	-59.54 (-37.02)
	Anti-parallel	-49.88 (-34.82)	-52.59 (-36.01)	-47.84 (-31.00)
$^{L}F_3{}^{D}K$	Parallel	-57.77 (-48.91)	-61.22 (-39.80)	-57.72 (-37.55)
	Anti-parallel	-51.10 (-36.12)	-54.16 (-36.03)	-41.16 (-33.81)
$^{D}F_3{}^{L}K$	Parallel	-57.43 (-38.10)	-56.14 (-38.48)	-51.97 (-31.39)
	Anti-parallel	-46.73 (-34.02)	-52.93 (-33.28)	-43.95 (-28.75)
$^{D}F_3{}^{D}K$	Parallel	-68.28 (-44.33)	-64.64 (-43.54)	-51.49 (-39.80)
	Anti-parallel	-48.53 (-38.06)	-50.06 (-38.98)	-48.50 (-33.91)
$^{D}I_3{}^{L}K$	Parallel	-49.59 (-29.45)	-55.21 (-32.94)	-40.73 (-24.99)
	Anti-parallel	-51.77 (-33.79)	-58.30 (-37.06)	-46.43(-30.78)
$^{D}I_3{}^{D}K$	Parallel	-53.42 (-34.38)	-53.15 (-33.11)	-45.94 (-30.85)
	Anti-parallel	-57.52 (-37.04)	-58.75 (-38.33)	-47.16 (-32.14)

Supplementary references

(1) Fuster, F. & Silvi, B. Does the topological approach characterize the hydrogen bond? *Theor. Chem. Acc.* **104**, 13−21 (2000).

(2) Wang, M. et al. Unexpected role of achiral glycine in determining the suprastructural handedness of peptide nanofibrils. *ACS Nano* **15**, 10328−10341 (2021).

(3) Plimpton, S. Fast parallel algorithms for short-range molecular dynamics. *J. Comput. Phys.* **117**, 1−19 (1995).

(4) Jorgensen, W. L. & Tirado-Rives, J. The OPLS [optimized potentials for liquid simulations] potential functions for proteins, energy minimizations for crystals of cyclic peptides and crambin. *J. Am. Chem. Soc.* **110**, 1657−1666 (1988).

(5) Jorgensen, W. L., Maxwell, D. S. & Tirado-Rives, J. Development and testing of the OPLS all-atom force field on conformational energetics and properties of organic liquids. *J. Am. Chem. Soc.* **118**, 11225−11236 (1996).

(6) Berendsen, H. J., Grigera, J. R. & Straatsma, T. P. The missing term in effective pair potentials. *J. Phys. Chem.* **91**, 6269−6271 (1987).

(7) Nosé, S. A unified formulation of the constant temperature molecular dynamics methods. *J. Chem. Phys.* **81**, 511−519 (1984).

(8) Martyna, G. J., Tobias, D. J. & Klein, M. L. Constant pressure molecular dynamics algorithms. *J. Chem. Phys.* **101**, 4177−4189 (1994).

(9) Hockney, R. W., Goel, S. & Eastwood, J. Quiet high-resolution computer models of a plasma. *J.*

Comput. Phys. **14**, 148−158 (1974).

(10) Humphrey, W., Dalke, A. & Schulten, K. VMD: visual molecular dynamics. *J. Mol. Graphics* **14**, 33−38 (1996).
